# Supplementary material for: Comparison between machine learning methods for mortality prediction for sepsis patients with different social determinants
Source: BMC Med Inform Decis Mak. 2022 Jun 16;22(Suppl 2):156. doi: 10.1186/s12911-022-01871-0 (PMC9204861; doi:10.1186/s12911-022-01871-0)
Supplement: Supplementary file 1 — Additional file 1. Supplementary tables for proportions of subpopulations, observed differences for sex, marital status, and insurance type groups, and pairwise comparisons among sex, marital status, and insurance type groups. [file 12911_2022_1871_MOESM1_ESM.docx]

Table S1. Proportions and confidence intervals for each subpopulation and criteria for 11,791 patients.

|  | Explicit | | | Angus | | | Martin | | | CMS | | | CDC | | | Sepsis-3 | | |
| --- | --- | --- | --- | --- | --- | --- | --- | --- | --- | --- | --- | --- | --- | --- | --- | --- | --- | --- |
| **Race** | **prop** | **lower** | **upper** | **prop** | **lower** | **upper** | **prop** | **lower** | **upper** | **prop** | **lower** | **upper** | **prop** | **lower** | **upper** | **prop** | **lower** | **upper** |
| Asian | 0.113 | 0.080 | 0.146 | 0.274 | 0.229 | 0.321 | 0.182 | 0.140 | 0.223 | 0.140 | 0.101 | 0.179 | 0.345 | 0.295 | 0.396 | 0.533 | 0.479 | 0.583 |
| Black or African American | 0.075 | 0.060 | 0.090 | 0.272 | 0.245 | 0.298 | 0.122 | 0.103 | 0.140 | 0.094 | 0.077 | 0.111 | 0.296 | 0.269 | 0.323 | 0.453 | 0.428 | 0.484 |
| Hispanic or Latino | 0.053 | 0.033 | 0.072 | 0.223 | 0.184 | 0.260 | 0.116 | 0.085 | 0.147 | 0.085 | 0.061 | 0.109 | 0.243 | 0.204 | 0.284 | 0.411 | 0.368 | 0.455 |
| Other | 0.098 | 0.084 | 0.113 | 0.322 | 0.298 | 0.347 | 0.157 | 0.139 | 0.176 | 0.117 | 0.101 | 0.133 | 0.373 | 0.349 | 0.400 | 0.511 | 0.486 | 0.538 |
| White | 0.092 | 0.086 | 0.098 | 0.285 | 0.276 | 0.295 | 0.149 | 0.142 | 0.157 | 0.112 | 0.105 | 0.118 | 0.316 | 0.307 | 0.326 | 0.494 | 0.484 | 0.505 |
| **Sex** |  |  |  |  |  |  |  |  |  |  |  |  |  |  |  |  |  |  |
| Female | 0.088 | 0.080 | 0.095 | 0.299 | 0.286 | 0.312 | 0.144 | 0.135 | 0.154 | 0.109 | 0.101 | 0.118 | 0.308 | 0.296 | 0.320 | 0.482 | 0.468 | 0.495 |
| Male | 0.092 | 0.085 | 0.099 | 0.275 | 0.265 | 0.286 | 0.149 | 0.140 | 0.158 | 0.111 | 0.103 | 0.119 | 0.328 | 0.316 | 0.339 | 0.497 | 0.485 | 0.510 |
| **Marital Status** |  |  |  |  |  |  | ` |  |  |  |  |  |  |  |  |  |  |  |
| Separated | 0.086 | 0.068 | 0.107 | 0.303 | 0.270 | 0.333 | 0.145 | 0.121 | 0.171 | 0.110 | 0.091 | 0.132 | 0.332 | 0.303 | 0.365 | 0.482 | 0.447 | 0.515 |
| Significant Other | 0.087 | 0.079 | 0.094 | 0.266 | 0.254 | 0.278 | 0.143 | 0.134 | 0.153 | 0.109 | 0.101 | 0.117 | 0.322 | 0.309 | 0.335 | 0.488 | 0.475 | 0.502 |
| Single | 0.088 | 0.079 | 0.098 | 0.287 | 0.271 | 0.302 | 0.148 | 0.136 | 0.160 | 0.104 | 0.093 | 0.114 | 0.294 | 0.279 | 0.310 | 0.477 | 0.460 | 0.494 |
| Unknown | 0.084 | 0.064 | 0.105 | 0.276 | 0.244 | 0.311 | 0.121 | 0.097 | 0.147 | 0.103 | 0.081 | 0.127 | 0.336 | 0.302 | 0.375 | 0.473 | 0.437 | 0.511 |
| Widowed | 0.111 | 0.095 | 0.125 | 0.343 | 0.322 | 0.366 | 0.170 | 0.151 | 0.187 | 0.134 | 0.117 | 0.151 | 0.347 | 0.323 | 0.369 | 0.539 | 0.514 | 0.565 |
| **Insurance type** |  |  |  |  |  |  |  |  |  |  |  |  |  |  |  |  |  |  |
| Government | 0.060 | 0.039 | 0.083 | 0.244 | 0.207 | 0.285 | 0.097 | 0.069 | 0.124 | 0.067 | 0.046 | 0.090 | 0.255 | 0.216 | 0.297 | 0.382 | 0.338 | 0.430 |
| Medicaid | 0.077 | 0.062 | 0.093 | 0.279 | 0.255 | 0.307 | 0.136 | 0.117 | 0.155 | 0.097 | 0.081 | 0.113 | 0.298 | 0.272 | 0.325 | 0.468 | 0.443 | 0.497 |
| Medicare | 0.108 | 0.101 | 0.117 | 0.333 | 0.321 | 0.344 | 0.173 | 0.164 | 0.183 | 0.134 | 0.126 | 0.144 | 0.353 | 0.341 | 0.365 | 0.548 | 0.535 | 0.561 |
| Private | 0.070 | 0.063 | 0.079 | 0.225 | 0.212 | 0.239 | 0.118 | 0.108 | 0.128 | 0.084 | 0.075 | 0.093 | 0.283 | 0.269 | 0.298 | 0.426 | 0.410 | 0.442 |
| Self-Pay | 0.037 | 0.012 | 0.068 | 0.111 | 0.068 | 0.160 | 0.068 | 0.037 | 0.105 | 0.056 | 0.025 | 0.093 | 0.198 | 0.136 | 0.259 | 0.309 | 0.241 | 0.377 |
| **Language** |  |  |  |  |  |  |  |  |  |  |  |  |  |  |  |  |  |  |
| English | 0.895 | 0.877 | 0.913 | 0.892 | 0.882 | 0.902 | 0.895 | 0.881 | 0.909 | 0.886 | 0.868 | 0.902 | 0.893 | 0.883 | 0.903 | 0.893 | 0.886 | 0.901 |
| Other | 0.095 | 0.078 | 0.112 | 0.088 | 0.079 | 0.098 | 0.088 | 0.074 | 0.102 | 0.098 | 0.083 | 0.115 | 0.087 | 0.078 | 0.097 | 0.086 | 0.079 | 0.094 |
| Spanish | 0.010 | 0.005 | 0.017 | 0.020 | 0.015 | 0.025 | 0.017 | 0.011 | 0.023 | 0.016 | 0.010 | 0.023 | 0.019 | 0.015 | 0.024 | 0.020 | 0.017 | 0.024 |

*The colors are the same as Figure 2 in the main text. prop: proportion; lower: lower bound of the 95% confidence interval; upper: the upper bound of the 95% confidence interval. Explicit: the explicit criteria; Angus: the Angus methodology; Martin: the Martine methodology; CMS: criteria presented by Centers for Medicare & Medicaid Services (CMS); CDC: the complete surveillance criteria presented by Center of Disease Control and Prevention (CDC); Sepsis-3: the Sepsis-3 criteria.*

Table S2. Observed differences between the testing results and each sex with p-values from permutation tests.

|  | **Female** | | **Male** | |
| --- | --- | --- | --- | --- |
|  | Observed difference | *p_val* | Observed difference | *p_val* |
| **Ridge classifier** | -0.0215 | *0.248* | 0.0168 | *0.294* |
| **Perceptron** | -0.0168 | *0.331* | 0.0137 | *0.331* |
| **Passive-aggressive** | -0.0262 | *0.231* | 0.0210 | *0.283* |
| **kNN** | -0.0363 | *0.148* | 0.0285 | *0.207* |
| **Random forest** | -0.0060 | *0.425* | 0.0046 | *0.437* |
| **LinearSVC_L1** | -0.0211 | *0.297* | 0.0165 | *0.313* |
| **LinearSVC_L2** | -0.0214 | *0.268* | 0.0167 | *0.335* |
| **SGDClassifier_L1** | -0.0185 | *0.317* | 0.0184 | *0.290* |
| **SGDClassifier_L2** | -0.0181 | *0.306* | 0.0194 | *0.270* |
| **SGDClassifier_EN** | -0.0192 | *0.316* | 0.0190 | *0.265* |
| **MultinomialNB** | -0.0046 | *0.449* | 0.0064 | *0.413* |
| **BernoulliNB** | 0.0017 | *0.496* | -0.0010 | *0.474* |
| **Logistic regression** | -0.0225 | *0.248* | 0.0176 | *0.297* |
| **SVC_rbf** | -0.0215 | *0.269* | 0.0163 | *0.324* |
| **SVC_poly** | -0.0190 | *0.304* | 0.0151 | *0.338* |
| **SVC_sigmoid** | -0.0210 | *0.260* | 0.0174 | *0.294* |

*Observe difference: observed difference in AUC when compared with the performance on the entire testing set; p_val: p-value, p-values less than or equal to 0.05 were highlighted; Passive-aggressive: passive-aggressive classifier; kNN: k-Nearest Neighbors; LinearSVC_L1 or _L2: support vector machine with linear kernel coupled with L1 or L2 regularization; SGDClassifier_L1 or _L2 or _EN: stochastic gradient descent with L1 or L2 or Elastic Net regularization; MultinomialNB: Multinomial naïve Bayes; BernoulliNB: Bernoulli naïve Bayes; SVC_rbf or _poly or _sigmoid: support vector machine with rbf kernel or polynomial kernel or sigmoid kernel.*

Table S3. Observed differences between the testing results and each marital status with p-values from permutation tests.

|  | **Separated** | | **Significant Other** | | **Single** | | **Unknown** | | **Widowed** | |
| --- | --- | --- | --- | --- | --- | --- | --- | --- | --- | --- |
|  | Observed difference | *p_val* | Observed difference | *p_val* | Observed difference | *p_val* | Observed difference | *p_val* | Observed difference | *p_val* |
| **Ridge classifier** | 0.0379 | *0.314* | 0.0056 | *0.469* | -0.0420 | *0.219* | -0.0622 | *0.215* | 0.0255 | *0.309* |
| **Perceptron** | 0.0351 | *0.310* | -0.0010 | *0.476* | -0.0252 | *0.296* | -0.0655 | *0.172* | 0.0171 | *0.380* |
| **Passive-aggressive** | 0.0044 | *0.457* | -0.0021 | *0.458* | -0.0550 | *0.152* | -0.0309 | *0.350* | 0.0502 | *0.145* |
| **kNN** | 0.0034 | *0.451* | 0.0003 | *0.487* | -0.0377 | *0.216* | -0.0923 | *0.090* | 0.0506 | *0.168* |
| **Random forest** | -0.0366 | *0.267* | -0.0007 | *0.499* | 0.0153 | *0.332* | -0.0900 | *0.074* | 0.0173 | *0.322* |
| **LinearSVC_L1** | 0.0395 | *0.306* | 0.0040 | *0.482* | -0.0371 | *0.221* | -0.0664 | *0.204* | 0.0223 | *0.327* |
| **LinearSVC_L2** | 0.0388 | *0.289* | 0.0050 | *0.451* | -0.0406 | *0.217* | -0.0660 | *0.213* | 0.0251 | *0.319* |
| **SGDClassifier_L1** | 0.0395 | *0.307* | 0.0043 | *0.491* | -0.0333 | *0.242* | -0.0656 | *0.197* | 0.0261 | *0.301* |
| **SGDClassifier_L2** | 0.0405 | *0.287* | 0.0054 | *0.438* | -0.0343 | *0.232* | -0.0660 | *0.188* | 0.0279 | *0.280* |
| **SGDClassifier_EN** | 0.0379 | *0.333* | 0.0041 | *0.432* | -0.0345 | *0.231* | -0.0651 | *0.209* | 0.0276 | *0.286* |
| **MultinomialNB** | 0.0015 | *0.516* | -0.0087 | *0.427* | 0.0134 | *0.367* | -0.0500 | *0.251* | 0.0341 | *0.244* |
| **BernoulliNB** | 0.0046 | *0.487* | 0.0026 | *0.466* | 0.0176 | *0.347* | -0.0496 | *0.179* | 0.0000 | *0.511* |
| **Logistic regression** | 0.0349 | *0.330* | 0.0061 | *0.429* | -0.0437 | *0.199* | -0.0651 | *0.183* | 0.0290 | *0.267* |
| **SVC_rbf** | 0.0289 | *0.338* | -0.0015 | *0.498* | -0.0325 | *0.261* | -0.0677 | *0.184* | 0.0228 | *0.320* |
| **SVC_poly** | 0.0436 | *0.289* | 0.0023 | *0.465* | -0.0364 | *0.232* | -0.0755 | *0.142* | 0.0218 | *0.339* |
| **SVC_sigmoid** | 0.0550 | *0.242* | 0.0359 | *0.152* | -0.0995 | ***0.030*** | -0.0611 | *0.211* | 0.0004 | *0.504* |

*Observe difference: observed difference in AUC when compared with the performance on the entire testing set; p_val: p-value, p-values less than or equal to 0.05 were highlighted; Passive-aggressive: passive-aggressive classifier; kNN: k-Nearest Neighbors; LinearSVC_L1 or _L2: support vector machine with linear kernel coupled with L1 or L2 regularization; SGDClassifier_L1 or _L2 or _EN: stochastic gradient descent with L1 or L2 or Elastic Net regularization; MultinomialNB: Multinomial naïve Bayes; BernoulliNB: Bernoulli naïve Bayes; SVC_rbf or _poly or _sigmoid: support vector machine with rbf kernel or polynomial kernel or sigmoid kernel.*

Table S4. Observed differences between the testing results and each insurance type with p-values from permutation tests.

|  | **Government** | | **Medicaid** | | **Medicare** | | **Private** | | **Self-Pay** | |
| --- | --- | --- | --- | --- | --- | --- | --- | --- | --- | --- |
|  | Observed difference | *p_val* | Observed difference | *p_val* | Observed difference | *p_val* | Observed difference | *p_val* | Observed difference | *p_val* |
| **Ridge classifier** | 0.0269 | *0.428* | 0.0074 | *0.484* | -0.0219 | *0.246* | 0.0192 | *0.364* | 0.1114 | *0.227* |
| **Perceptron** | 0.0214 | *0.427* | 0.0129 | *0.441* | -0.0251 | *0.214* | 0.0257 | *0.273* | 0.1103 | *0.239* |
| **Passive-aggressive** | 0.0591 | *0.341* | -0.0065 | *0.448* | -0.0138 | *0.332* | 0.0220 | *0.336* | 0.1677 | *0.159* |
| **kNN** | 0.0810 | *0.307* | -0.0305 | *0.326* | -0.0178 | *0.246* | 0.0170 | *0.360* | 0.1498 | *0.180* |
| **Random forest** | 0.0693 | *0.282* | 0.0230 | *0.323* | -0.0126 | *0.293* | 0.0027 | *0.453* | 0.0578 | *0.299* |
| **LinearSVC_L1** | 0.0263 | *0.430* | 0.0085 | *0.458* | -0.0223 | *0.234* | 0.0206 | *0.333* | 0.1108 | *0.271* |
| **LinearSVC_L2** | 0.0266 | *0.426* | 0.0075 | *0.462* | -0.0223 | *0.238* | 0.0194 | *0.343* | 0.1112 | *0.265* |
| **SGDClassifier_L1** | 0.0285 | *0.426* | 0.0107 | *0.460* | -0.0208 | *0.256* | 0.0226 | *0.319* | 0.1130 | *0.246* |
| **SGDClassifier_L2** | 0.0295 | *0.431* | 0.0086 | *0.445* | -0.0201 | *0.251* | 0.0229 | *0.329* | 0.1140 | *0.244* |
| **SGDClassifier_EN** | 0.0334 | *0.428* | 0.0076 | *0.434* | -0.0205 | *0.265* | 0.0220 | *0.346* | 0.1136 | *0.233* |
| **MultinomialNB** | 0.0265 | *0.402* | 0.0397 | *0.285* | -0.0220 | *0.258* | 0.0711 | *0.073* | 0.1479 | *0.179* |
| **BernoulliNB** | -0.0289 | *0.400* | -0.0185 | *0.371* | -0.0029 | *0.468* | 0.0171 | *0.350* | -0.0168 | *0.426* |
| **Logistic regression** | 0.0326 | *0.392* | 0.0039 | *0.456* | -0.0206 | *0.246* | 0.0166 | *0.379* | 0.1128 | *0.232* |
| **SVC_rbf** | -0.0048 | *0.495* | 0.0036 | *0.487* | -0.0220 | *0.256* | 0.0243 | *0.307* | 0.0960 | *0.279* |
| **SVC_poly** | 0.0423 | *0.380* | 0.0004 | *0.512* | -0.0201 | *0.276* | 0.0161 | *0.390* | 0.1508 | *0.151* |
| **SVC_sigmoid** | 0.1127 | *0.195* | -0.0137 | *0.412* | 0.0053 | *0.413* | -0.0369 | *0.209* | -0.0021 | *0.508* |

*Observe difference: observed difference in AUC when compared with the performance on the entire testing set; p_val: p-value, p-values less than or equal to 0.05 were highlighted; Passive-aggressive: passive-aggressive classifier; kNN: k-Nearest Neighbors; LinearSVC_L1 or _L2: support vector machine with linear kernel coupled with L1 or L2 regularization; SGDClassifier_L1 or _L2 or _EN: stochastic gradient descent with L1 or L2 or Elastic Net regularization; MultinomialNB: Multinomial naïve Bayes; BernoulliNB: Bernoulli naïve Bayes; SVC_rbf or _poly or _sigmoid: support vector machine with rbf kernel or polynomial kernel or sigmoid kernel.*

Table S5. Pairwise comparisons among different sex groups.

|  | **Female v.s. Male** | |
| --- | --- | --- |
|  | Observed difference | *p_val* |
| **Ridge classifier** | 0.0384 | *0.324* |
| **Perceptron** | 0.0305 | *0.444* |
| **Passive-aggressive** | 0.0471 | *0.220* |
| **kNN** | 0.0648 | *0.096* |
| **Random forest** | 0.0106 | *0.698* |
| **LinearSVC_L1** | 0.0376 | *0.331* |
| **LinearSVC_L2** | 0.0381 | *0.364* |
| **SGDClassifier_L1** | 0.0368 | *0.390* |
| **SGDClassifier_L2** | 0.0375 | *0.320* |
| **SGDClassifier_EN** | 0.0382 | *0.340* |
| **MultinomialNB** | 0.0110 | *0.783* |
| **BernoulliNB** | -0.0027 | *0.921* |
| **Logistic regression** | 0.0402 | *0.330* |
| **SVC_rbf** | 0.0378 | *0.324* |
| **SVC_poly** | 0.0340 | *0.386* |
| **SVC_sigmoid** | 0.0384 | *0.325* |

*Observe difference: observed difference in AUC when comparing the performance between the sub-populations; p_val: p-value, p-values less than or equal to 0.05 were highlighted; Passive-aggressive: passive-aggressive classifier; kNN: k-Nearest Neighbors; LinearSVC_L1 or _L2: support vector machine with linear kernel coupled with L1 or L2 regularization; SGDClassifier_L1 or _L2 or _EN: stochastic gradient descent with L1 or L2 or Elastic Net regularization; MultinomialNB: Multinomial naïve Bayes; BernoulliNB: Bernoulli naïve Bayes; SVC_rbf or _poly or _sigmoid: support vector machine with rbf kernel or polynomial kernel or sigmoid kernel.*

Table S6. Pairwise comparisons among different marital status groups.

|  | **Seperated v.s. Significant Other** | | **Seperated v.s. Single** | | **Seperated v.s. Unknown** | | **Seperated v.s. Widowed** | | **Significant Other v.s. Single** | |
| --- | --- | --- | --- | --- | --- | --- | --- | --- | --- | --- |
|  | Observed difference | *p_val* | Observed difference | *p_val* | Observed difference | *p_val* | Observed difference | *p_val* | Observed difference | *p_val* |
| **Ridge classifier** | -0.0324 | *0.695* | -0.0800 | *0.339* | -0.1001 | *0.316* | -0.0124 | *0.889* | -0.0476 | *0.393* |
| **Perceptron** | -0.0361 | *0.645* | -0.0603 | *0.486* | -0.1006 | *0.321* | -0.0180 | *0.814* | -0.0242 | *0.659* |
| **Passive-aggressive** | -0.0065 | *0.936* | -0.0594 | *0.501* | -0.0353 | *0.748* | 0.0458 | *0.582* | -0.0529 | *0.351* |
| **kNN** | -0.0031 | *0.974* | -0.0410 | *0.619* | -0.0957 | *0.338* | 0.0472 | *0.572* | -0.0379 | *0.458* |
| **Random forest** | 0.0359 | *0.538* | 0.0519 | *0.416* | -0.0534 | *0.479* | 0.0539 | *0.463* | 0.0160 | *0.679* |
| **LinearSVC_L1** | -0.0355 | *0.667* | -0.0766 | *0.335* | -0.1059 | *0.284* | -0.0172 | *0.822* | -0.0411 | *0.462* |
| **LinearSVC_L2** | -0.0337 | *0.695* | -0.0793 | *0.324* | -0.1048 | *0.339* | -0.0136 | *0.897* | -0.0456 | *0.395* |
| **SGDClassifier_L1** | -0.0352 | *0.649* | -0.0728 | *0.365* | -0.1051 | *0.302* | -0.0134 | *0.890* | -0.0376 | *0.480* |
| **SGDClassifier_L2** | -0.0351 | *0.650* | -0.0748 | *0.386* | -0.1065 | *0.300* | -0.0126 | *0.879* | -0.0397 | *0.448* |
| **SGDClassifier_EN** | -0.0338 | *0.623* | -0.0724 | *0.415* | -0.1029 | *0.315* | -0.0103 | *0.908* | -0.0386 | *0.473* |
| **MultinomialNB** | -0.0103 | *0.902* | 0.0119 | *0.891* | -0.0515 | *0.596* | 0.0326 | *0.715* | 0.0222 | *0.689* |
| **BernoulliNB** | -0.0020 | *0.965* | 0.0131 | *0.816* | -0.0541 | *0.349* | -0.0046 | *0.936* | 0.0151 | *0.653* |
| **Logistic regression** | -0.0287 | *0.732* | -0.0786 | *0.347* | -0.1000 | *0.328* | -0.0059 | *0.954* | -0.0498 | *0.360* |
| **SVC_rbf** | -0.0304 | *0.705* | -0.0614 | *0.495* | -0.0966 | *0.334* | -0.0061 | *0.943* | -0.0310 | *0.582* |
| **SVC_poly** | -0.0413 | *0.574* | -0.0800 | *0.372* | -0.1191 | *0.231* | -0.0218 | *0.806* | -0.0386 | *0.479* |
| **SVC_sigmoid** | -0.0191 | *0.824* | -0.1545 | *0.054* | -0.1161 | *0.274* | -0.0546 | *0.487* | -0.1354 | ***0.022*** |
|  | **Significant Other v.s. Unknown** | | **Significant Other v.s. Widowed** | | **Single v.s. Unknown** | | **Single v.s. Widowed** | | **Unknown v.s. Widowed** | |
|  | Observed difference | *p_val* | Observed difference | *p_val* | Observed difference | *p_val* | Observed difference | *p_val* | Observed difference | *p_val* |
| **Ridge classifier** | -0.0678 | *0.356* | 0.0199 | *0.722* | -0.0202 | *0.811* | 0.0676 | *0.283* | 0.0877 | *0.300* |
| **Perceptron** | -0.0645 | *0.403* | 0.0181 | *0.725* | -0.0404 | *0.621* | 0.0422 | *0.511* | 0.0826 | *0.344* |
| **Passive-aggressive** | -0.0288 | *0.742* | 0.0523 | *0.320* | 0.0242 | *0.778* | 0.1052 | *0.112* | 0.0811 | *0.349* |
| **kNN** | -0.0926 | *0.206* | 0.0503 | *0.349* | -0.0546 | *0.523* | 0.0883 | *0.165* | 0.1429 | *0.081* |
| **Random forest** | -0.0893 | *0.143* | 0.0180 | *0.676* | -0.1053 | *0.096* | 0.0020 | *0.966* | 0.1073 | *0.141* |
| **LinearSVC_L1** | -0.0704 | *0.339* | 0.0183 | *0.774* | -0.0293 | *0.731* | 0.0594 | *0.364* | 0.0887 | *0.283* |
| **LinearSVC_L2** | -0.0711 | *0.344* | 0.0201 | *0.712* | -0.0255 | *0.765* | 0.0657 | *0.314* | 0.0912 | *0.278* |
| **SGDClassifier_L1** | -0.0699 | *0.344* | 0.0217 | *0.690* | -0.0323 | *0.735* | 0.0594 | *0.371* | 0.0917 | *0.304* |
| **SGDClassifier_L2** | -0.0714 | *0.380* | 0.0225 | *0.683* | -0.0317 | *0.714* | 0.0622 | *0.308* | 0.0940 | *0.272* |
| **SGDClassifier_EN** | -0.0692 | *0.363* | 0.0235 | *0.644* | -0.0306 | *0.700* | 0.0620 | *0.347* | 0.0926 | *0.263* |
| **MultinomialNB** | -0.0413 | *0.588* | 0.0428 | *0.418* | -0.0634 | *0.450* | 0.0207 | *0.758* | 0.0841 | *0.338* |
| **BernoulliNB** | -0.0521 | *0.316* | -0.0026 | *0.947* | -0.0672 | *0.206* | -0.0177 | *0.676* | 0.0495 | *0.398* |
| **Logistic regression** | -0.0713 | *0.358* | 0.0228 | *0.676* | -0.0215 | *0.795* | 0.0727 | *0.286* | 0.0941 | *0.255* |
| **SVC_rbf** | -0.0662 | *0.381* | 0.0243 | *0.680* | -0.0352 | *0.656* | 0.0553 | *0.382* | 0.0905 | *0.282* |
| **SVC_poly** | -0.0778 | *0.291* | 0.0195 | *0.728* | -0.0391 | *0.635* | 0.0582 | *0.391* | 0.0973 | *0.265* |
| **SVC_sigmoid** | -0.0971 | *0.195* | -0.0355 | *0.509* | 0.0384 | *0.632* | 0.0999 | *0.126* | 0.0615 | *0.471* |

*Observe difference: observed difference in AUC when comparing the performance between the sub-populations; p_val: p-value, p-values less than or equal to 0.05 were highlighted; Passive-aggressive: passive-aggressive classifier; kNN: k-Nearest Neighbors; LinearSVC_L1 or _L2: support vector machine with linear kernel coupled with L1 or L2 regularization; SGDClassifier_L1 or _L2 or _EN: stochastic gradient descent with L1 or L2 or Elastic Net regularization; MultinomialNB: Multinomial naïve Bayes; BernoulliNB: Bernoulli naïve Bayes; SVC_rbf or _poly or _sigmoid: support vector machine with rbf kernel or polynomial kernel or sigmoid kernel.*

Table S7. Pairwise comparisons among different insurance type groups.

|  | **Government v.s. Medicaid** | | **Government v.s. Medicare** | | **Government v.s. Private** | | **Government v.s. Self Pay** | | **Medicaid v.s. Medicare** | |
| --- | --- | --- | --- | --- | --- | --- | --- | --- | --- | --- |
|  | Observed difference | *p_val* | Observed difference | *p_val* | Observed difference | *p_val* | Observed difference | *p_val* | Observed difference | *p_val* |
| **Ridge classifier** | -0.0195 | *0.895* | -0.0488 | *0.732* | -0.0077 | *0.955* | 0.0845 | *0.693* | -0.0294 | *0.670* |
| **Perceptron** | -0.0085 | *0.946* | -0.0465 | *0.737* | 0.0043 | *0.982* | 0.0889 | *0.674* | -0.0379 | *0.604* |
| **Passive-aggressive** | -0.0657 | *0.672* | -0.0729 | *0.600* | -0.0371 | *0.817* | 0.1085 | *0.608* | -0.0072 | *0.918* |
| **kNN** | -0.1115 | *0.469* | -0.0988 | *0.467* | -0.0640 | *0.626* | 0.0688 | *0.744* | 0.0127 | *0.862* |
| **Random forest** | -0.0463 | *0.692* | -0.0819 | *0.490* | -0.0665 | *0.562* | -0.0115 | *0.957* | -0.0356 | *0.534* |
| **LinearSVC_L1** | -0.0177 | *0.911* | -0.0486 | *0.729* | -0.0057 | *0.968* | 0.0845 | *0.702* | -0.0308 | *0.645* |
| **LinearSVC_L2** | -0.0192 | *0.899* | -0.0489 | *0.734* | -0.0072 | *0.957* | 0.0845 | *0.691* | -0.0297 | *0.651* |
| **SGDClassifier_L1** | -0.0177 | *0.909* | -0.0492 | *0.766* | -0.0058 | *0.972* | 0.0845 | *0.710* | -0.0315 | *0.649* |
| **SGDClassifier_L2** | -0.0209 | *0.891* | -0.0496 | *0.725* | -0.0066 | *0.963* | 0.0845 | *0.693* | -0.0287 | *0.665* |
| **SGDClassifier_EN** | -0.0258 | *0.849* | -0.0539 | *0.704* | -0.0114 | *0.941* | 0.0802 | *0.724* | -0.0281 | *0.681* |
| **MultinomialNB** | 0.0133 | *0.924* | -0.0485 | *0.731* | 0.0447 | *0.764* | 0.1214 | *0.576* | -0.0617 | *0.363* |
| **BernoulliNB** | 0.0104 | *0.912* | 0.0260 | *0.772* | 0.0460 | *0.643* | 0.0121 | *0.826* | 0.0155 | *0.744* |
| **Logistic regression** | -0.0287 | *0.842* | -0.0532 | *0.713* | -0.0160 | *0.902* | 0.0802 | *0.722* | -0.0245 | *0.719* |
| **SVC_rbf** | 0.0085 | *0.961* | -0.0171 | *0.901* | 0.0292 | *0.860* | 0.1008 | *0.614* | -0.0256 | *0.724* |
| **SVC_poly** | -0.0419 | *0.763* | -0.0624 | *0.668* | -0.0262 | *0.862* | 0.1085 | *0.618* | -0.0204 | *0.757* |
| **SVC_sigmoid** | -0.1264 | *0.423* | -0.1074 | *0.465* | -0.1496 | *0.299* | -0.1148 | *0.598* | 0.0190 | *0.798* |
|  | **Medicaid v.s. Private** | | **Medicaid v.s. Self Pay** | | **Medicare v.s. Private** | | **Medicare v.s. Self Pay** | | **Private v.s. Self Pay** | |
|  | Observed difference | *p_val* | Observed difference | *p_val* | Observed difference | *p_val* | Observed difference | *p_val* | Observed difference | *p_val* |
| **Ridge classifier** | 0.0117 | *0.864* | 0.1040 | *0.561* | 0.0411 | *0.379* | 0.1334 | *0.377* | 0.0923 | *0.583* |
| **Perceptron** | 0.0128 | *0.852* | 0.0974 | *0.585* | 0.0507 | *0.317* | 0.1353 | *0.412* | 0.0846 | *0.598* |
| **Passive-aggressive** | 0.0285 | *0.700* | 0.1742 | *0.339* | 0.0358 | *0.493* | 0.1814 | *0.271* | 0.1457 | *0.359* |
| **kNN** | 0.0475 | *0.547* | 0.1803 | *0.293* | 0.0348 | *0.440* | 0.1675 | *0.303* | 0.1327 | *0.411* |
| **Random forest** | -0.0202 | *0.728* | 0.0348 | *0.734* | 0.0154 | *0.675* | 0.0704 | *0.597* | 0.0550 | *0.646* |
| **LinearSVC_L1** | 0.0121 | *0.884* | 0.1023 | *0.560* | 0.0429 | *0.407* | 0.1331 | *0.432* | 0.0902 | *0.589* |
| **LinearSVC_L2** | 0.0120 | *0.890* | 0.1037 | *0.572* | 0.0417 | *0.405* | 0.1334 | *0.405* | 0.0918 | *0.593* |
| **SGDClassifier_L1** | 0.0119 | *0.879* | 0.1023 | *0.544* | 0.0434 | *0.371* | 0.1338 | *0.417* | 0.0904 | *0.604* |
| **SGDClassifier_L2** | 0.0143 | *0.852* | 0.1054 | *0.557* | 0.0430 | *0.392* | 0.1342 | *0.422* | 0.0911 | *0.592* |
| **SGDClassifier_EN** | 0.0144 | *0.852* | 0.1060 | *0.518* | 0.0425 | *0.365* | 0.1341 | *0.420* | 0.0916 | *0.585* |
| **MultinomialNB** | 0.0314 | *0.708* | 0.1081 | *0.556* | 0.0931 | *0.059* | 0.1699 | *0.314* | 0.0767 | *0.622* |
| **BernoulliNB** | 0.0355 | *0.524* | 0.0016 | *0.978* | 0.0200 | *0.553* | -0.0139 | *0.906* | -0.0339 | *0.748* |
| **Logistic regression** | 0.0127 | *0.874* | 0.1089 | *0.523* | 0.0372 | *0.458* | 0.1334 | *0.402* | 0.0962 | *0.580* |
| **SVC_rbf** | 0.0207 | *0.786* | 0.0923 | *0.594* | 0.0463 | *0.372* | 0.1179 | *0.493* | 0.0716 | *0.669* |
| **SVC_poly** | 0.0158 | *0.838* | 0.1505 | *0.363* | 0.0362 | *0.459* | 0.1709 | *0.301* | 0.1347 | *0.428* |
| **SVC_sigmoid** | -0.0233 | *0.753* | 0.0116 | *0.948* | -0.0423 | *0.373* | -0.0075 | *0.964* | 0.0348 | *0.825* |

*Observe difference: observed difference in AUC when comparing the performance between the sub-populations; p_val: p-value, p-values less than or equal to 0.05 were highlighted; Passive-aggressive: passive-aggressive classifier; kNN: k-Nearest Neighbors; LinearSVC_L1 or _L2: support vector machine with linear kernel coupled with L1 or L2 regularization; SGDClassifier_L1 or _L2 or _EN: stochastic gradient descent with L1 or L2 or Elastic Net regularization; MultinomialNB: Multinomial naïve Bayes; BernoulliNB: Bernoulli naïve Bayes; SVC_rbf or _poly or _sigmoid: support vector machine with rbf kernel or polynomial kernel or sigmoid kernel.*
